# Improving the repeatability of deep learning models with Monte Carlo dropout

**DOI:** 10.1038/s41746-022-00709-3

**Published:** 2022-11-18

**Authors:** Andreanne Lemay, Katharina Hoebel, Christopher P. Bridge, Brian Befano, Silvia De Sanjosé, Didem Egemen, Ana Cecilia Rodriguez, Mark Schiffman, John Peter Campbell, Jayashree Kalpathy-Cramer

**Affiliations:** 1 Martinos Center for Biomedical Imaging, Boston, MA USA; 2 NeuroPoly, Polytechnique Montreal, Montreal, QC Canada; 3 Massachusetts Institute of Technology, Cambridge, MA USA; 4 MGH & BWH Center for Clinical Data Science, Boston, MA USA; 5 Department of Epidemiology, University of Washington School of Public Health, Seattle, WA USA; 6 Division of Cancer Epidemiology & Genetics, National Cancer Institute, Rockville, MD USA; 7 Oregon Health and Science University, Portland, OR USA

**Keywords:** Biomedical engineering, Medical imaging

## Abstract

The integration of artificial intelligence into clinical workflows requires reliable and robust models. Repeatability is a key attribute of model robustness. Ideal repeatable models output predictions without variation during independent tests carried out under similar conditions. However, slight variations, though not ideal, may be unavoidable and acceptable in practice. During model development and evaluation, much attention is given to classification performance while model repeatability is rarely assessed, leading to the development of models that are unusable in clinical practice. In this work, we evaluate the repeatability of four model types (binary classification, multi-class classification, ordinal classification, and regression) on images that were acquired from the same patient during the same visit. We study the each model’s performance on four medical image classification tasks from public and private datasets: knee osteoarthritis, cervical cancer screening, breast density estimation, and retinopathy of prematurity. Repeatability is measured and compared on ResNet and DenseNet architectures. Moreover, we assess the impact of sampling Monte Carlo dropout predictions at test time on classification performance and repeatability. Leveraging Monte Carlo predictions significantly increases repeatability, in particular at the class boundaries, for all tasks on the binary, multi-class, and ordinal models leading to an average reduction of the 95% limits of agreement by 16% points and of the class disagreement rate by 7% points. The classification accuracy improves in most settings along with the repeatability. Our results suggest that beyond about 20 Monte Carlo iterations, there is no further gain in repeatability. In addition to the higher test-retest agreement, Monte Carlo predictions are better calibrated which leads to output probabilities reflecting more accurately the true likelihood of being correctly classified.

## Introduction

**Fig. 1:**
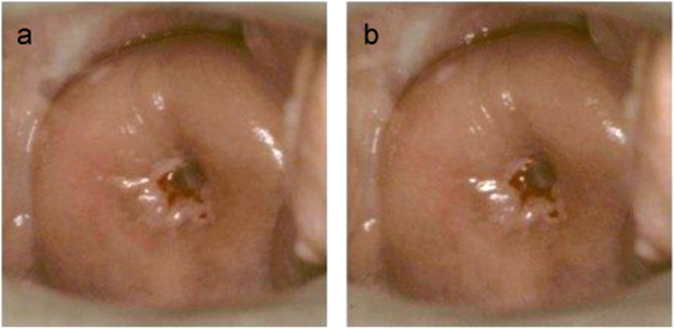
Illustration of repeatability issues from deep learning models on different images of a cervix with precancerous lesions from the same patient taken the same day. A binary model without dropout layers generated the following outputs. (**a**) Model prediction: 0.01 (Normal): The binary model predicts a normal cervix (severity score: 0.01). (**b**) Model prediction: 0.98 (Pre-cancer): The binary model predicts pre-cancer (severity score: 0.98).

Deep learning is a popular technology to achieve high performance for medical image analysis tasks. In the desire to achieve higher classification performance, important aspects of the model performance, such as test-retest variability remain overlooked, yet not all deep learning (DL) models are equal with respect to their repeatability. Consistency in the prediction of models is of utmost importance for such models to prove their potential as reliable and safe clinical support. However, DL models face substantial repeatability issues^1,2^. Empirically, minor changes in an image can lead to vastly different predictions by DL models. In clinical practice, this repeatability issue could lead to dangerous medical errors. Figure 1 illustrates this issue. Two cervical cancer screening images from the same precancerous cervix that were taken during the same visit led to completely different predictions. A binary DL model (without dropout layers) trained to distinguish between a normal cervix and one with a precancerous lesion (0: Normal, 1: Pre-cancer) predicted a normal cervix on one image and classified the second image as precancerous. This difference is represented by prediction results at each extreme of the spectrum, i.e., 0.01 and 0.98, suggesting high certainty for both outputs.

Dropout is the process of randomly removing units from a neural network during training to regularize learning and avoid overfitting^3,4^. For inference, dropout is usually disabled to leverage all the connections from the model. Gal et al.^5^ proposed to enable dropout at test time as a Bayesian approximation to sample multiple different predictions. From these Monte Carlo (MC) predictions, it is possible to derive uncertainty metrics that are indicative of model performance^6^ which has already been explored for multiple medical image classification tasks^7–9^. The final prediction is usually generated by taking the average overall MC predictions. We will refer to these models utilizing dropout as *MC models*.

Repeatability describes the variation between independent tests taken under the same conditions. In this work, we focus on repeatability of a single model using different images of the same anatomical region from the same patient taken the same day. For the public knee osteoarthritis dataset, only one image per knee for a given time point was available, hence, a second image was generated using minor data augmentation. To the best of our knowledge, few studies focus on methodologies to increase repeatability. However, some work notes the importance of repeatability for medical image analysis by assessing the test-retest reliability of their classification or segmentation models^2,10–15^. Kim et al.^2^ evaluated the test-retest variability for disease classification on chest radiographs and obtained limits of agreement (LoA) of ± 30% indicating variability within the test-retest predictions. Various post-processing techniques such as blurring or sharpening, which could naturally occur in real-life settings and alter the appearance of images, caused higher test-retest variability compared to positional changes. Multiple other factors have been shown to impact repeatability such as inter-rater variability in the labels, image quality, noise, or model uncertainty due to lack of knowledge and limited number of images, i.e., epistemic uncertainty^2,16^. For instance, images leading to high inter-rater variability among experts are likely to generate similar variability, especially at class boundaries^17^, since the model was trained based on the ratings of these experts. While some of these factors leading to low repeatability cannot be eliminated in practice (e.g., inter-rater variability), reliable DL models should be robust to minor changes in position, lighting, focus, etc.

Calibrated models will output probabilities reflecting the probability of the observed outcome (e.g., all the predictions of 0.9 from a perfectly calibrated model should have the positive class as ground truth 90% of the time). Good calibration allows robust rejection of low probability predictions as output probabilities represent more truthfully the likelihood of being wrong. Modern neural networks are poorly calibrated due to the recent neural network advances in architecture and training^18^. Multiple works have focused on developing methods for post-hoc calibration of models^18–20^ usually taking the validation set to adjust the test prediction. However, having an inherently more calibrated output could mitigate the need for prediction re-calibration. Brier score is a common metric to assess calibration as it indicates how close the predicted probabilities are to the true likelihood. A Brier score of 0 indicates perfect calibration.

Repeatability is an important and required characteristic of medial image analysis tools as it reflects the ability of the model to repeatedly generate a certain classification performance. More repeatable models with the same accuracy provide smaller variability in accuracy for a single measurement per patient. Hence, repeatable models generate more consistent classification performance leading to less variability.

While most works describing the development of DL models for medical image classification focus on accuracy and classification performance^21–23^, very few assess the repeatability of these models. This study proposes Monte Carlo (MC) dropout at test time as a method to improve repeatability and systematically assess this approach on different tasks, model types, and network architectures. All the selected medical tasks have an underlying continuous scale of disease severity but are routinely binned into binary or ordinal classes to simplify treatment decisions and ratings. Although specifically training networks to assess disease severity might be a preferred approach^24–26^, this is rarely done in practice^27–29^. The methodology and analysis were chosen based on the consideration that the underlying variable of interest, i.e., disease severity, of these medical tasks is better represented by a spectrum rather than clear distinct categories. In this work, we evaluate the model repeatability of four types of DL models, binary classification, multiclass classification, ordinal classification, and regression, each with and without MC dropout. We test the repeatability of these models’ predictions on four different medical image classification tasks: knee osteoarthritis grading, cervical cancer screening, breast density estimation, and retinopathy of prematurity (ROP) disease severity grading. True test-retest scenarios are studied with private datasets containing multiple images per patient for a given time point and anatomical region. Few public datasets exist with multiple images from the same anatomical region taken during the same visit. As we acknowledge the importance of reproducibility in research, a fourth dataset that is publicly available, the Multicenter Osteoarthritis Study, was added to the study and a second image per patient was generated by applying simple data augmentation to the original image, i.e., horizontal flip, to simulate test-retest reliability. Based on our results, we present recommendations for model choices that can lead to improved repeatability. Finally, we assess the calibration of regular models compared to MC models.

## Results

### Repeatability and classification performance

**Fig. 2:**
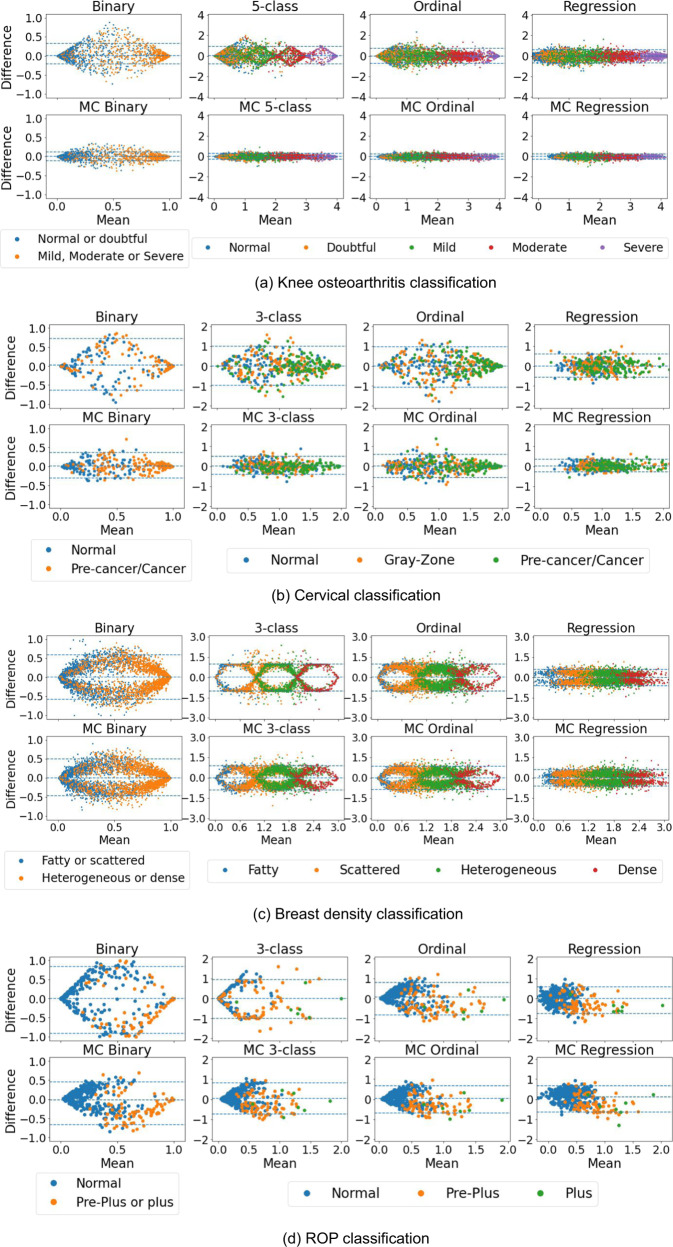
Bland-Altman plots on multiple images from the same patient and visit. The y-axis of each graph represents the maximum difference in model prediction for images of the same patient, while the x-axis refers to the mean of the predicted scores. (**a**) Knee osteoarthritis, (**b**) cervical, (**c**) breast density, and (**d**) ROP classification. The 95% limits of agreement are presented with dashed blue lines. Repeatable models are associated with differences and limits of agreement closer to zero which indicates a smaller difference between test and retest.

The repeatability of each model was assessed on all available images of the same patient during the same visit. MC dropout models were associated with increased repeatability and accuracy for all models and tasks excluding regression models (Table 1 and Fig. 2). Bland-Altman plots for all the tasks and model types are summarized in Fig. 2. An alternative way to compare the severity score from the test and retest images is presented in Supplementary Information. Ideally, all cases would lie near a horizontal line crossing the y-axis at 0 which means the difference between test-retest scores is low. For every task, the MC models showed better test-retest reliability than their conventional counterparts with the exception of the regression models. This is illustrated by the narrower 95% LoA and the highest concentration of differences near 0 on the y-axis. Model outputs exhibit higher differences near class boundaries. However, this effect is attenuated for MC models and almost absent for regression models. The range of predicted values remained similar for MC models, indicating that the effect of the MC model is not simply regressing scores towards the mean. Moreover, the increase in repeatability was in most cases associated with an improvement in classification performance (Table 1).

**Table 1:** Model performance overview (MEAN ± 95% CI)^a^.

	Repeatability metrics		Classification metrics	
Model	Disag. rate *↓*	95% LoA *↓*	*κ* *↑*	Acc. *↑*
**Knee osteoarthritis classification**				
Binary	0.05 ± 0.01	0.27 ± 0.02	0.87 ± 0.01	0.95 ± 0.00
MC Bin.	**0.02** ± **0.00**	**0.11** ± **0.01**	**0.89** ± **0.01**	**0.95** ± **0.00**
5-class	0.25 ± 0.01	0.22 ± 0.01	0.88 ± 0.01	0.69 ± 0.01
MC 5-cl.	**0.10** ± **0.01**	**0.07** ± **0.00**	**0.91** ± **0.00**	**0.72** ± **0.01**
Ord.	0.15 ± 0.01	0.19 ± 0.01	0.84 ± 0.01	0.54 ± 0.01
MC ord.	**0.08** ± **0.01**	**0.07** ± **0.00**	**0.85** ± **0.01**	**0.56** ± **0.01**
Reg.	0.19 ± 0.01	0.16 ± 0.00	**0.90** ± **0.00**	**0.70** ± **0.01**
MC Reg.	**0.14** ± **0.01**	**0.07** ± **0.00**	0.88 ± 0.00	0.61 ± 0.01
**Cervical classification**				
Binary	0.23 ± 0.05	0.68 ± 0.07	0.46 ± 0.07	0.73 ± 0.03
MC Bin.	**0.13** ± **0.04**	**0.33** ± **0.04**	**0.51** ± **0.07**	**0.75** ± **0.03**
3-class	0.38 ± 0.05	0.50 ± 0.06	0.34 ± 0.06	0.47 ± 0.03
MC 3-cl.	**0.24** ± **0.04**	**0.22** ± **0.03**	**0.42** ± **0.06**	**0.52** ± **0.03**
Ord.	0.37 ± 0.05	0.51 ± 0.07	0.38 ± 0.06	0.47 ± 0.03
MC ord.	**0.28** ± **0.04**	**0.29** ± **0.03**	**0.41** ± **0.06**	**0.49** ± **0.03**
Reg.	0.31 ± 0.04	0.29 ± 0.03	0.34 ± 0.05	**0.44** ± **0.03**
MC Reg.	**0.19** ± **0.04**	**0.16** ± **0.02**	**0.35** ± **0.05**	0.43 ± 0.03
**Breast density classification**				
Binary	0.22 ± 0.01	0.58 ± 0.01	0.68 ± 0.01	0.84 ± 0.00
MC Bin.	**0.19** ± **0.01**	**0.48** ± **0.01**	**0.69** ± **0.01**	**0.85** ± **0.00**
4-class	0.54 ± 0.02	0.33 ± 0.00	0.71 ± 0.01	0.69 ± 0.01
MC 4-cl.	**0.45** ± **0.01**	**0.30** ± **0.00**	**0.72** ± **0.01**	**0.71** ± **0.01**
Ord.	0.52 ± 0.01	0.33 ± 0.00	0.70 ± 0.01	0.68 ± 0.01
MC ord.	**0.44** ± **0.01**	**0.29** ± **0.01**	**0.72** ± **0.01**	**0.69** ± **0.01**
Reg.	**0.39** ± **0.01**	0.21 ± 0.01	0.74 ± 0.01	**0.70** ± **0.01**
MC Reg.	0.40 ± 0.01	0.21 ± 0.01	**0.75** ± **0.01**	0.67 ± 0.01
**ROP classification**				
Binary	0.31 ± 0.01	0.88 ± 0.04	0.50 ± 0.05	0.81 ± 0.02
MC Bin.	**0.25** ± **0.04**	**0.55** ± **0.05**	**0.56** ± **0.05**	**0.85** ± **0.02**
3-class	0.23 ± 0.04	0.48 ± 0.03	**0.57** ± **0.06**	0.85 ± 0.02
MC 3-cl.	0.23 ± 0.04	**0.39** ± **0.03**	0.55 ± 0.06	**0.85** ± **0.02**
Ord.	0.31 ± 0.04	0.40 ± 0.04	0.57 ± 0.05	0.82 ± 0.02
MC ord.	**0.29** ± **0.04**	**0.34** ± **0.03**	0.57 ± 0.05	**0.83** ± **0.02**
Reg.	**0.16** ± **0.04**	**0.33** ± **0.03**	**0.58** ± **0.06**	**0.86** ± **0.02**
MC Reg.	0.47 ± 0.05	0.33 ± 0.01	0.51 ± 0.05	0.79 ± 0.03

^a^ Values in bold indicate the best model between MC and non-MC models where a statistical difference (*p* *value* > 0.05) was observed. The two first columns measure the model repeatability where smaller values indicate better repeatability. The two last columns represent the model performance and high values indicate better classification. Binary models were trained with the following classes: Knee osteoarthritis: none and doubtful vs. mild, moderate, and severe – Cervix: normal vs. pre-cancer/cancer – Breast density: fatty and scattered vs. heterogeneous and dense – ROP: normal vs. pre-plus and plus. *LoA* Limits of agreement; *κ*: Quadratic weighted Cohen’s *κ*; *Acc*. Accuracy; *CI* Confidence interval.

**Fig. 3:**
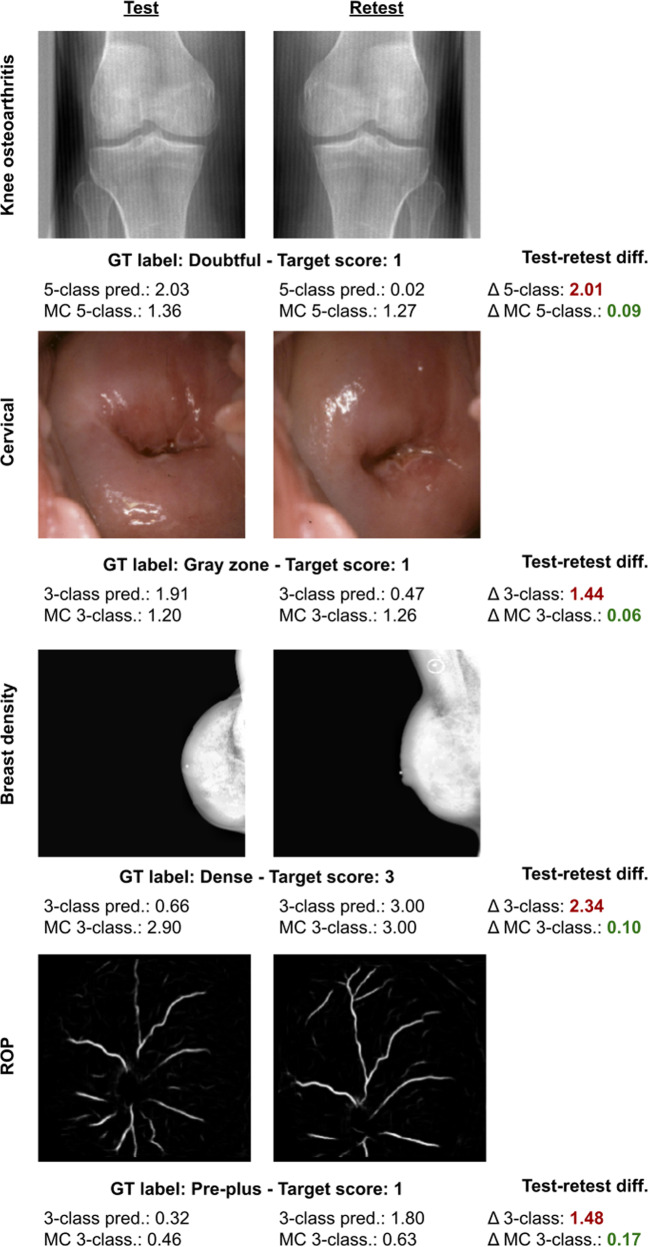
Repeatability comparison for multi-class vs. MC multi-class models for each dataset. These cases illustrate extreme cases where multi-class predictions failed to be repeatable while MC multi-class performed decently. These examples were picked choosing images where the repeatability difference between the continuous scores was the largest between multi-class and MC multiclass.

Repeatability and classification metrics for each approach can be found in Table 1. Repeatability of MC models for binary, multi-class, and ordinal models showed statistically significant improvements on at least one metric for all tasks. On average, across all tasks and classification models (i.e., excluding regression), the disagreement rate improved by 7% points and the 95% limit of the agreement by 16% points. Classification performance followed the same trend as the repeatability and increased for all classification MC models with the exception of the ROP task, which was exposed to a domain shift (see Discussion). Figure 3 illustrates cases where multiclass models showed poor repeatability while the MC multi-class was significantly more repeatable. While there are minor differences between test and retest images, we expect the model to be robust to changes in view, lighting, or zoom, as the disease severity or breast density does not change from one image to another. Adding MC iterations to regression models did not lead to consistent improvement in classification or repeatability performances. Regression models generally showed better repeatability compared to the other multi-class models (i.e., n-class and ordinal).

### Impact of number of MC iterations

**Fig. 4:**
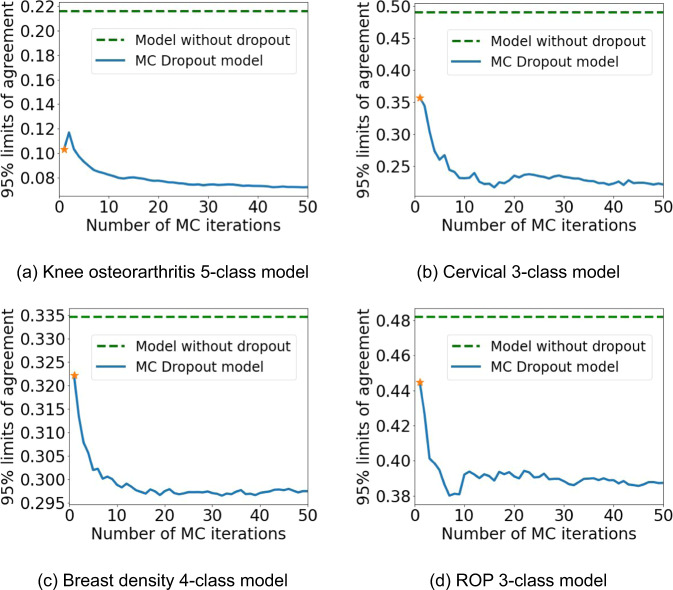
Impact of number of MC iterations on repeatability. (**a**) Knee osteoarthritis 5-class model. (**b**) Cervical 3-class model. (**c**) Breast density 4-class model. (**d**) ROP 3-class model. The orange star represents a single forward pass of the model with dropout disabled at test time.

Additionally, we evaluated the impact of increasing the number of MC iterations at test time to compute the final prediction on repeatability of MC models, i.e., 95% LoA, of multi-class models for all tasks as illustrated in Fig. 4. This analysis was limited to the multi-class models as they are the most commonly used for medical classification tasks. All models suggest that training with dropout, even without any MC iterations during testing, has better test-retest performance than non-dropout models (Fig. 4). Repeatability could be further improved by generating more MC samples. After about 20 MC iterations, additional samples had little to no impact on repeatability.

### Architecture comparison

**Fig. 5:**
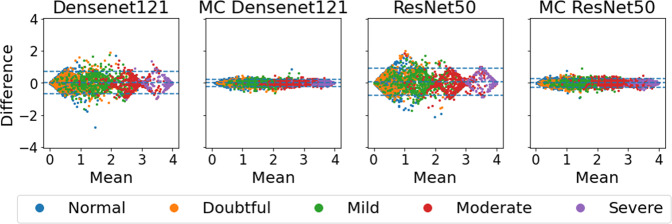
Architecture comparison on multiclass model for knee osteoarthritis grading. The two first columns are the model trained with Densenet121, while the two last ones were trained with ResNet50. The first and third graphs represent the regular model and the second and fourth ones display their MC counterparts.

Figure 5 compares, for the same task (i.e., knee osteoarthritis grading) and model type (i.e., multi-class), the DenseNet and ResNet architectures with respect to repeatability. Regardless of the model’s architecture, the behavior remains the same: the test-retest variability is lower meaning repeatability is increased when using multiple MC samples for the prediction. The disagreement rate decreased of 9% and 15% points and the LoA improved by 11% and 15% points for DenseNet and ResNet architectures, respectively.

### Calibration

**Fig. 6:**
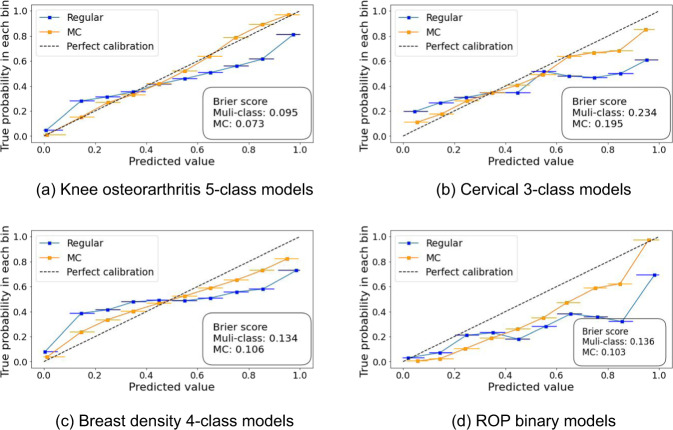
Calibration curves. (**a**) Knee osteoarthritis 5-class models. (**b**) Cervical 3-class models. (**c**) Breast density 4-class models. (**d**) ROP binary models. Brier score quantifies model calibration: 0 indicates a perfectly calibrated model. The horizontal bars represent the predicted value distribution (95% CI) for every bin.

Output probabilities are more calibrated for MC models than for the regular models as depicted in Fig. 6. Brier scores associated with MC models are lower for all tasks, i.e., average decrease of 0.031, and the calibration curves are closer to the identity line, i.e., the perfect calibration curve. Calibration curves of multi-class model outputs were displayed for knee osteoarthritis, cervix and breast density classification, while the binary models were chosen for ROP as the impact of adding MC was greater for this task compared with the multi-class models (see Table 1).

## Discussion

Our results demonstrate that MC dropout models lead to a significant increase in repeatability, i.e., improvement of at least one repeatability metric, while improving most classification metrics for binary, multi-class, and ordinal models. Concretely, this means higher class and score agreements between the test and retest outputs. The repeatability increased regardless of the disease imaged or the model architecture (DenseNet or ResNet). However, MC iterations did not benefit all regression models and even lowered classification performance for knee osteoarthritis and ROP classification. Regression models showed higher repeatability compared with non-MC multi-class and ordinal models, so the potential gain was more modest. For the two datasets where MC dropout did not improve repeatability on the regression model, i.e., cervical and ROP datasets, the highest repeatability was already reached by the regression model. Hence, in these cases, the models might have reached a limit in repeatability where MC dropout is of no extra help. While the lowest test-retest variability was reached for the regression model on the knee and cervical images, the model was associated with a lower quadratic *κ* and/or accuracy. Both accuracy and repeatability need to be reported to thoroughly assess deep learning models, especially in clinical settings.

The observed differences between test-retest images of the same patient were not constant along the mean axis as seen on the Bland-Altman plots in Fig. 2. Near the class boundaries, images show more variability with only a few cases with a difference near zero, which creates an arch-like pattern in the plots. MC dropout models stands out by its ability to improve repeatability at the class boundaries where non MC models display more oscillation patterns between classes. Non-MC models tend to avoid ambivalent class predictions to the benefit of choosing one class creating poor repeatability at class boundaries. When the model is equivocal about the class, MC dropout models have a better ability to output the same prediction score. This phenomenon can be partly explained by the training scheme of classification models. During training, models are optimized to predict classes with high certainty, discouraging the model from outputting ambivalent predictions (e.g., predicting 0.5 for a binary model), which leads to uncalibrated models^18^. Ideally, the output softmax or sigmoid probability of a model should reflect the uncertainty of the model between two or more classes. However, in practice, this is not the case leading to high differences in the class boundaries due to the misclassification of at least one of the images. This effect is alleviated with MC models, leading to more calibrated outputs and higher repeatability.

Fewer repeatability metrics showed a statistical difference between MC dropout and conventional models for the ROP disease severity classification task. Unlike knee osteoarthritis, cervical, and breast density classification, the ROP models were tested on views of the eye that the model has not seen during training (section Retinopathy of Prematurity). This domain shift might be adding variability in the model’s prediction impacting the global performance and repeatability, effectively abating the benefits of MC dropout models. Nonetheless, MC models still showed higher repeatability under domain shift than no-dropout models.

MC models are computationally more expensive than their conventional counterparts as they require multiple forward passes at testing time. Our results in Fig. 4 indicate that after approximately 20 MC iterations, there is no further gain in repeatability, and this, for all tasks on multi-class models. For settings where time and computational resources are limited, training with dropout layers, even without sampling multiple MC, helps regularize the training and reduces overfitting^3^.

Due to the high number of model types studied (8) and datasets (4), each model was trained only once. Varying data splittings for the training, validation, and test sets could help get a sense of the variability of the metrics for each model type. The analysis is limited to ResNet and DenseNet architectures for classification. Other architectures could behave differently with MC dropout. Future studies should focus on more extensive model architectures for classification and segmentation tasks. Finally, all the medical tasks studied in this work are prone to inter-rater variability. However, not all labels from the knee osteoarthritis, cervical, and breast density datasets were derived from multiple experts, which can affect the models’ performance.

We evaluated the repeatability of four model types on four medical tasks using distinct model architectures (ResNet18, ResNet50, DenseNet121). We demonstrated that MC sampling during test time leads to more reliable models providing more stable, repeatable, and calibrated predictions on different images from the same patient with or without a slight domain shift. MC dropout models reduced test-retest variability at the class boundaries where repeatability is the most challenging and crucial. Only regression models did not show a constant improvement when leveraging MC sampling. Repeatability metrics increased with an increasing number of MC iterations; after around 20 MC iterations, no further improvement of repeatability could be reached. MC sampling is flexible as it is applicable to any model type and architecture while being easily implementable. Future work should assess the impact of MC models on repeatability for other model architectures and other tasks such as segmentation.

## Methods

All images were de-identified prior to data access, ethical approval for this study was therefore not required.

### Knee osteoarthritis

Knee osteoarthritis is the most common musculoskeletal disorder^30^ and was the eleventh-highest contributor to global disability in 2010^31^. Osteoarthritis can be diagnosed with a radiography, however, early diagnosis can be challenging in clinical practice and is prone to inter-rater variability justifying the emergence of AI models for osteoarthritis grading^30^. The severity is typically measured using the Kellgren-Lawrence (KL) scale from 0 to 4 where 0 corresponds to none, 1 to doubtful, 2 to mild, 3 to moderate, and 4 to severe^32^.

The publicly available longitudinal Multicenter Osteoarthritis Study (MOST) dataset contains 18,926 knee radiographs from 3017 patients of one or both knees when including only grades from 0 to 4 on the Kellgren-Lawrence scale^32^. Grades outside the Kellgren-Lawrence scale were excluded from the dataset for this work. 40% of the cases were labeled as grade 0, 15% as grade 1, 17% as grade 2, 19% as grade 3, and 9% as grade 4. The patients were split into training, validation, and test sets representing 65%, 10%, and 25% of the images, respectively. The binary models were trained to distinguish between knees with no or doubtful osteoarthritis (negative class) and knees with mild, moderate, or severe osteoarthritis (positive class). Images were center cropped to a size of 224x224 pixels and scaled to intensity values of 0 to 1. MOST does not include multiple images of the same during the same visit. Model predictions were generated for all the original test images, were then flipped horizontally, and retested to emulate a test-retest setting. Hence, the repeatability was measured on the same radiography from the same patient at a given time point with and without the horizontal flip. Since flipping is applied as data augmentation during training, we expect all models to be robust to this affine transformation (see Section Classification model training for details on training data augmentation).

### Cervical

Cervical cancer is the fourth most common cancer worldwide and the leading cause of cancer-related deaths of women in western, eastern, middle, and southern Africa^33^. Vaccinations against high-risk strains of the Human Papilloma Virus (HPV) have been proven to prevent up to 90% of cervical cancers^34^. Until HPV vaccination programs have not reached every eligible woman worldwide, and in light of the high prevalence of high-risk HPV types, there will be a great demand for effective screening at low costs to prevent the development of invasive cervical cancer. In addition to HPV testing, the visual assessment of the cervix using photographs can help to detect precancerous lesions in low-resource settings^35–37^.

The cervical cancer screening dataset consisted of 3509 cervical photographs from 1760 patients from two studies^38,39^. For most patients, we had access to two cervical photographs taken during the same session.

Each image was classified using cytological and histological data from the patient as one of the following three categories: Normal (1148 images, 33%), Gray zone, i.e., the presence of precancerous lesions was equivocal, (1159 images, 33%), Precancer/cancer (1202 images, 34%).

The dataset was split into training (65%), validation (10%), and test sets (25%) on a patient level, resulting in datasets containing 2283, 350, and 876 images (training/validation/test), preserving the class distributions described above within each subset. All images were de-identified before this study. All cervical images were cropped using bounding boxes from a trained Retina net for cervix detection, resized to 256x256 pixels, and scaled to intensity values of 0 to 1. The cervigram classification models were trained using all photographs for each patient in the training dataset. For the binary classification models, we utilized only images that were classified as either normal or pre-cancer/cancer. For all patients in the test dataset for whom both images were available, repeatability was assessed as the difference in predictions between the two photographs.

### Breast density

Breast cancer is the second most common cause of cancer deaths among women in the USA with an estimated number of more than 41,000 deaths in 2019^40^. The density of a women’s breast is determined by the amount of fibroglandular tissue. It can be classified (with increasing density) based on its appearance on x-ray mammography as almost entirely fatty, scattered fibroglandular densities, heterogeneously dense, and extremely dense^41^. Importantly, the risk of developing breast cancer rises with increasing breast density^42^. Furthermore^43^, have shown that women with extremely dense breast tissue benefit from additional MRI screening. The development of AI models based on expert labels for breast density assessment could help to mitigate intra-, and interobserver variability and the inconsistency of current quantitative measurements with expert raters^28^.

The Digital Mammographic Imaging Screening Trial (DMIST) dataset consists of a total of 108,230 mammograms from 21,729 patients acquired at 33 institutions with an average of five mammographs of different standard mammography views for each patient^44^. Breast density labels were generated according to the BI-RADS criteria^41^ by a total of 92 different radiologists. The dataset consisted of 12,428 (11.5%) fatty, 47,909 (44.2%) scattered, 41,325 (38.2%) heterogeneously dense, and 6568 (6.1%) extremely dense samples and was split into training (70,293), validation (10,849), and test datasets (27,048 images) on a patient level preserving the label distribution of the full dataset. All images were de-identified before this study. We cropped all images to a size of 224x224 pixels. The breast density classification models were trained using all available views for each patient in the training dataset using either four labels or a simplified binary labeling system of fatty and scattered as one class, and dense and heterogeneous as the other class. Repeatability was assessed as the maximum difference between all available views for each patient in the test dataset.

### Retinopathy of prematurity

ROP is the leading cause of preventable childhood blindness worldwide^45^. It gets diagnosed based on the appearance of the retinal vessel tree on retinal photographs and classified into three discrete disease severity classes: normal, pre-plus, and plus disease^46^. However, the disease spectrum is continuous^26^ and the use of discrete class labels to train DL classifiers is complicated by inter-rater variability particularly for cases close to the class boundaries^17,47^. High interrater variability, an insufficient number of ophthalmologists and neonatologists with the expertise and willingness (e.g., due to significant malpractice liability) to manage ROP, and the rising incidence of ROP worldwide motivate the development of AI models for ROP classification and screening^48^.

The ROP dataset consists of 5511 retinal photographs acquired at eight different study centers^48^. For each patient, retinal photographs were acquired in 5 different standard fields of view (posterior, nasal, temporal, inferior, superior). Only the posterior, temporal, and nasal views were used in this study. Images were classified as normal, preplus disease, or plus disease following previously published methods^49^. The final label is based on the independent image-based diagnosis by 3 expert graders in combination with the full clinical diagnosis by an expert ophthalmologist. Of the 5511 images in the dataset, 4535 (82.3%) were classified as normal, 804 (14.6%) as pre-plus disease, and 172 (3.1%) as plus disease. The binary models were trained to distinguish between normal and pre-plus/plus disease. The dataset was split on a patient level into training, validation, and test datasets containing 4322/722/467 images while preserving the overall class distribution within each subset. Following^48^’s work, we trained ROP classification models using normalized pre-segmented vessel maps as input (size of 480x640). ROP classification models were trained using only the posterior field of view as ROP refers to arterial tortuosity and venous dilation within the posterior pole of the retina^50^. However, it was shown that experts use characteristics beyond the posterior view to assess ROP severity^50^. Hence, repeatability was tested using the posterior, temporal, and nasal views of all patients in the test dataset.

### Classification model training

For each dataset, we trained binary, multi-class, and ordinal^51^ classification models, as well as regression models each with and without dropout, resulting in a total of 8 models per dataset. We used the following ImageNet pretrained models for each dataset based on which performed the best for the conventional multi-class classification model: DenseNet121 (cervix), ResNet50 (knee osteoarthritis, breast density), and ResNet18 (ROP). Models were trained using binary cross-entropy, cross-entropy, CORAL^51^, and mean squared error (MSE) losses for binary, multi-class, ordinal, and regression models, respectively. Affine transformations, i.e., rotation ± 15 degrees and random horizontal flips with 50% probability, were applied as data augmentation during training. The code was implemented using the MONAI framework (version 0.5.2)^52^ based on the PyTorch library (version 1.9.0)^53^.

Models with dropout were trained using spatial dropout with a dropout rate of 0.1 for cervical images and DMIST, and 0.2 for knee osteoarthritis and ROP. The dropout rates were determined based on preliminary explorations to optimize the model’s classification performance and values from the literature^54–56^. Channels are independently and randomly zeroed for each dropout layer and forward pass, following the dropout rate from a Bernoulli distribution. For the DenseNet121 architecture, the dropout layers were applied after every dense layer, while for the ResNets the dropout layers were applied after each residual block. At test time, the dropout was enabled to generate *N* = 50 slightly different predictions and the final prediction was obtained by averaging over all the MC samples^5^. The choice of the number of MC predictions was based on values commonly found in the appropriate literature and experience; however, the optimal number of predictions to reach maximum repeatability was assessed in the results section (see Fig. 4).

This section enumerates the training parameters associated with the different datasets. These parameters were obtained by referring to previous work on these datasets^29,57,58^ or by initial dataset exploration. All models were trained with an Adam optimizer^59^ and a learning rate scheduler reducing of a factor 0.1 with a patience of 10 epochs. Table 2 enumerates the main training parameters that were different from a dataset to another.

**Table 2:** Overview of training parameters by dataset.

Datasets	Training parameters			
	Learning rate	Batch size	# epochs	Dropout rate
Knee osteoarthritis	5e-6	16	75	0.2
Cervical	1e-5	8	75	0.1
Breast density	5e-5	8	75	0.1
ROP	1e-4	24	25	0.2

### Evaluation

For direct comparison of a model’s predictions, we summarized each model’s outputs as a continuous severity score. For the binary and regression models, the output of the models was directly used without further modifications. For the multi-class model, we utilized the ordinality of all four classification problems and defined the continuous severity score as a weighted average using softmax probability of each class as described in Equation (1). For knee osteoarthritis (5 classes), the values lie in the range of 0 to 4, for breast density (4 classes) in the range of 0 to 3, and for cervical and ROP classification (3 classes), in the range of 0 to 2.

1 $$score=\mathop{\sum }\limits_{i=1}^{k}{p}_{i}\times i-1$$

with *k* being the number of classes and *p*_*i*_ the softmax probability of class *i*. For the ordinal model, the classification problem of *k* ranks (i.e., class) is modified into a *k* − 1 binary classification^60^ leading to one output unit less than for the traditional classification model. For instance, for a 3-class problem, the ground truth would be encoded as followed: class 1 → [0, 0]; class 2 → [1, 0]; class 3 → [1, 1]. The continuous prediction score for ordinal models is obtained by summing the output neurons. Similarly to the multi-class models, values range from 0 to 2, 0 to 3, and 0 to 4, for 3-class, 4-class, and 5-class problems, respectively.

Repeatability was evaluated using the classification disagreement rate and the 95% LoA from the Bland-Altman plots. Since normality was not reached for the differences for the LoA, non-parametric LoA were calculated using empirical percentiles^61^. The LoA was presented as a fraction of the possible value range. The classification disagreement rate corresponds to the proportion of patients with different classification outcomes for different images acquired during the same session over the total number of patients. The classification accuracy and quadratic weighted Cohen’s *κ* were also reported. For the regression models, thresholds to binarize predictions for accuracy and Cohen’s *κ* calculation were computed by splitting the range of predictions equally (e.g., 3-class problem: *s* ≤ 0.67 → class 1; 0.67 < *s* ≤ 1.33 → class 2; *s* ≥ 1.33 → class 3). Model calibration was assessed using the Brier score.

Statistical difference between models was determined using a two-sided *t*-test and metric bootstrapping (500 iterations). Models with a *p* value smaller than 0.05 were considered significantly different. The normality of the distribution was verified using the Shapiro-Wilk test (*α* = 0.05).

### Reporting summary

Further information on research design is available in the Nature Research Reporting Summary linked to this article.

## Supplementary information

Supplementary Information

Reporting Summary

## Data Availability

Access to the MOST dataset for knee osteoarthritis can be requested through the NIA Aging Research Biobank https://agingresearchbiobank.nia.nih.gov/. The cervical, breast density, and ROP datasets are not publicly accessible due to patient privacy restrictions.

## Code availability

The code used to train and generate results can be found at https://github.com/andreanne-lemay/gray_zone_assessment.

## References

[1] Alahmari SS, Goldgof DB, Mouton PR, Hall LO (2020). Challenges for the repeatability of deep learning models. IEEE Access.

[2] Kim H, Park CM, Goo JM (2020). Test-retest reproducibility of a deep learning–based automatic detection algorithm for the chest radiograph. Eur Radiol..

[3] Hinton, G. E., Srivastava, N., Krizhevsky, A., Sutskever, I. & Salakhutdinov, R. R. Improving neural networks by preventing co-adaptation of feature detectors. *arXiv preprint arXiv:1207.0580* (2012).

[4] Srivastava N, Hinton G, Krizhevsky A, Sutskever I, Salakhutdinov R (2014). Dropout: a simple way to prevent neural networks from overfitting. J. Mach. Learn. Res..

[5] Gal, Y. & Ghahramani, Z. Dropout as a bayesian approximation: Representing model uncertainty in deep learning. In *international conference on machine learning*, 1050–1059 (PMLR, 2016).

[6] Camarasa, R. et al. Quantitative comparison of monte-carlo dropout uncertainty measures for multi-class segmentation. In *Uncertainty for Safe Utilization of Machine Learning in Medical Imaging, and Graphs in Biomedical Image Analysis*, 32–41 (Springer, 2020).

[7] Leibig C, Allken V, Ayhan MS, Berens P, Wahl S (2017). Leveraging uncertainty information from deep neural networks for disease detection. Sci. Rep..

[8] Combalia, M., Hueto, F., Puig, S., Malvehy, J. & Vilaplana, V. Uncertainty estimation in deep neural networks for dermoscopic image classification. In *Proceedings of the IEEE/CVF Conference on Computer Vision and Pattern Recognition Workshops*, 744–745 (2020).

[9] Singh, R. K., Gorantla, R., Allada, S. G. R., & Narra, P. SkiNet: A deep learning framework for skin lesion diagnosis with uncertainty estimation and explainability. *Plos one*, **17**, e0276836 (2022). 10.1371/journal.pone.0276836 PMC9621459 36315487

[10] Hiremath A (2021). Test-retest repeatability of a deep learning architecture in detecting and segmenting clinically significant prostate cancer on apparent diffusion coefficient (adc) maps. Eur. Radiol..

[11] Estrada S (2020). Fatsegnet: A fully automated deep learning pipeline for adipose tissue segmentation on abdominal dixon mri. Magn. Reson. Med..

[12] Cole JH (2017). Predicting brain age with deep learning from raw imaging data results in a reliable and heritable biomarker. NeuroImage.

[13] Hoebel KV (2020). Radiomics repeatability pitfalls in a scan-rescan mri study of glioblastoma. Radiol.: Artif. Intell..

[14] Schwier M (2019). Repeatability of multiparametric prostate mri radiomics features. Sci. Rep..

[15] van Velden FH (2016). Repeatability of radiomic features in non-small-cell lung cancer [18 f] fdg-pet/ct studies: impact of reconstruction and delineation. Mol. Imag. Biol..

[16] Mojtahed, A. et al. Repeatability and reproducibility of deep-learning-based liver volume and couinaud segment volume measurement tool. *Abdominal Radiol.* 1–9 (2021). 10.1007/s00261-021-03262-x PMC8776724 34605963

[17] Kalpathy-Cramer J (2016). Plus Disease in Retinopathy of Prematurity: Improving Diagnosis by Ranking Disease Severity and Using Quantitative Image Analysis. Ophthalmology.

[18] Guo, C., Pleiss, G., Sun, Y. & Weinberger, K. Q.On calibration of modern neural networks. In *International Conference on Machine Learning*, 1321–1330 (PMLR, 2017).

[19] Kuleshov, V., Fenner, N. & Ermon, S. Accurate uncertainties for deep learning using calibrated regression. In *International Conference on Machine Learning*, 2796–2804 (PMLR, 2018).

[20] Laves, M.-H., Ihler, S., Fast, J. F., Kahrs, L. A. & Ortmaier, T. Well-calibrated regression uncertainty in medical imaging with deep learning. In *Medical Imaging with Deep Learning*, 393–412 (PMLR, 2020).

[21] Haenssle HA (2018). Man against machine: diagnostic performance of a deep learning convolutional neural network for dermoscopic melanoma recognition in comparison to 58 dermatologists. Ann. Oncol..

[22] Rajpurkar P (2018). Deep learning for chest radiograph diagnosis: A retrospective comparison of the chexnext algorithm to practicing radiologists. PLoS Med..

[23] Bakas, S., Reyes, M., Jakab, A., Bauer, S., Rempfler, M., Crimi, A., Shinohara, R. T., et al. Identifying the Best Machine Learning Algorithms for Brain Tumor Segmentation, Progression Assessment, and Overall Survival Prediction in the BRATS Challenge. 10.17863/CAM.38755 (2018).

[24] Li MD (2020). Siamese neural networks for continuous disease severity evaluation and change detection in medical imaging. NPJ Digital Med..

[25] Heine JJ, Cao K, Rollison DE, Tiffenberg G, Thomas JA (2011). A quantitative description of the percentage of breast density measurement using full-field digital mammography. Acad. Radiol..

[26] Campbell JP (2016). Plus Disease in Retinopathy of Prematurity: A Continuous Spectrum of Vascular Abnormality as a Basis of Diagnostic Variability. Ophthalmology.

[27] Thomas KA (2020). Automated classification of radiographic knee osteoarthritis severity using deep neural networks. Radiol.: Artif. Intell..

[28] Lehman CD (2019). Mammographic breast density assessment using deep learning: clinical implementation. Radiology.

[29] Brown JM (2018). Automated diagnosis of plus disease in retinopathy of prematurity using deep convolutional neural networks. JAMA Ophthalmol..

[30] Tiulpin A, Thevenot J, Rahtu E, Lehenkari P, Saarakkala S (2018). Automatic knee osteoarthritis diagnosis from plain radiographs: a deep learning-based approach. Sci. Rep..

[31] Cross M (2014). The global burden of hip and knee osteoarthritis: estimates from the global burden of disease 2010 study. Ann. Rheumatic Dis..

[32] Kellgren JH, Lawrence J (1957). Radiological assessment of osteo-arthrosis. Ann. Rheumatic Dis..

[33] Arbyn M (2020). Estimates of incidence and mortality of cervical cancer in 2018: a worldwide analysis. Lancet Global Health.

[34] Lei J (2020). HPV Vaccination and the Risk of Invasive Cervical Cancer. N. Engl. J. Med..

[35] Catarino R, Petignat P, Dongui G, Vassilakos P (2015). Cervical cancer screening in developing countries at a crossroad: Emerging technologies and policy choices. World J. Clin. Oncol..

[36] Xue Z (2020). A demonstration of automated visual evaluation of cervical images taken with a smartphone camera. Int. J. Cancer.

[37] Hu L (2019). An Observational Study of Deep Learning and Automated Evaluation of Cervical Images for Cancer Screening. J. Natl. Cancer Instit..

[38] Bratti MC (2004). Description of a seven-year prospective study of human papillomavirus infection and cervical neoplasia among 10 000 women in guanacaste, costa rica. Revista Panamericana de Salud Pública.

[39] Schiffman M, Solomon D (2003). Findings to date from the ascus-lsil triage study (alts). Arch. Pathol. Lab. Med..

[40] Siegel RL, Miller KD, Jemal A (2019). Cancer statistics, 2019. CA: A Cancer J. Clin..

[41] Liberman, L. & Menell, J. H.Breast imaging reporting and data system (BI-RADS) https://pubmed.ncbi.nlm.nih.gov/12117184/. (2002). 10.1016/s0033-8389(01)00017-3 12117184

[42] Boyd NF (1995). Quantitative classification of mammographic densities and breast cancer risk: Results from the canadian national breast screening study. J. Natl. Cancer Instit..

[43] Bakker MF (2019). Supplemental MRI Screening for Women with Extremely Dense Breast Tissue. N. Eng. J. Med..

[44] Pisano ED (2005). Diagnostic Performance of Digital versus Film Mammography for Breast-Cancer Screening. N. Eng. J. Med..

[45] IAPB, International Agency for the Prevention of Blindness. https://www.iapb.org:8443 (NA).

[46] Quinn GE (2005). The international classification of retinopathy of prematurity revisited: An international committee for the classification of retinopathy of prematurity. Arch. Ophthalmol..

[47] Chiang MF, Jiang L, Gelman R, Du YE, Flynn JT (2007). Interexpert agreement of plus disease diagnosis in retinopathy of prematurity. Arch. Ophthalmol..

[48] Brown JM (2018). Automated diagnosis of plus disease in retinopathy of prematurity using deep convolutional neural networks. JAMA Ophthalmol..

[49] Ryan MC (2014). Development and Evaluation of Reference Standards for Image-based Telemedicine Diagnosis and Clinical Research Studies in Ophthalmology. AMIA. Ann. Symp. Proc..

[50] Campbell JP (2016). Expert diagnosis of plus disease in retinopathy of prematurity from computer-based image analysis. JAMA Ophthalmol..

[51] Cao, W., Mirjalili, V., & Raschka, S. Rank consistent ordinal regression for neural networks with application to age estimation. *Pattern Recognit Lett*, **140**, 325–331 (2020).

[52] Consortium, T. M. Project monai (2020). 10.5281/zenodo.4323059.

[53] Paszke, A. et al. Pytorch: An imperative style, high-performance deep learning library. In Wallach, H. et al. (eds.) *Advances in Neural Information Processing Systems 32*, 8024-8035 (Curran Associates, Inc., 2019). http://papers.neurips.cc/paper/9015-pytorch-an-imperative-style-high-performance-deep-learning-library.pdf.

[54] Lévy, D. & Jain, A. Breast mass classification from mammograms using deep convolutional neural networks. In *Proceedings of the 30th Conference on Neural Information Processing Systems (NIPS 2016), Barcelona, Spain* (2016).

[55] Siddiqi, R. Automated pneumonia diagnosis using a customized sequential convolutional neural network. In *Proceedings of the 2019 3rd international conference on deep learning technologies*, 64–70 (2019).

[56] Sodmann P, Vollmer M, Nath N, Kaderali L (2018). A convolutional neural network for ecg annotation as the basis for classification of cardiac rhythms. Physiol. Measure..

[57] Li MD (2020). Siamese neural networks for continuous disease severity evaluation and change detection in medical imaging. npj Dig. Med..

[58] Chang K (2020). Multi-Institutional Assessment and Crowdsourcing Evaluation of Deep Learning for Automated Classification of Breast Density. J. Am. College .Radiol..

[59] Kingma, D., & Ba, J. Adam: A Method for Stochastic Optimization. *International Conference on Learning Representations* (2014).

[60] Li, L., & Lin, H. T. Ordinal regression by extended binary classification. *Advances in neural information processing systems* **19** (2006).

[61] Bland JM, Altman DG (1999). Measuring agreement in method comparison studies. Stat. Methods Med. Res..

