# Supplementary material for: Improving the repeatability of deep learning models with Monte Carlo dropout
Source: NPJ Digit Med. 2022 Nov 18;5:174. doi: 10.1038/s41746-022-00709-3 (PMC9674698; doi:10.1038/s41746-022-00709-3)
Supplement: Supplementary file 1 — Supplementary Information [file 41746_2022_709_MOESM1_ESM.pdf]

Supplementary Information

Test retest severity score visualization

An alternative way to present the data is to directly plot the severity score from a test image compare to the prediction obtained during retest. Supplementary Figure 1 displays the relation between pairs of images from the same patient taken at a given time point for all model types for knee osteoarthritis and cervical classification. When more than two images were available, the pair associated with the largest difference was selected. The data points are expected to lie near the identity line where the severity scores are equal. MC models exhibit increased repeatability compared with non-MC models as the data points are more concentrated near the identity line.

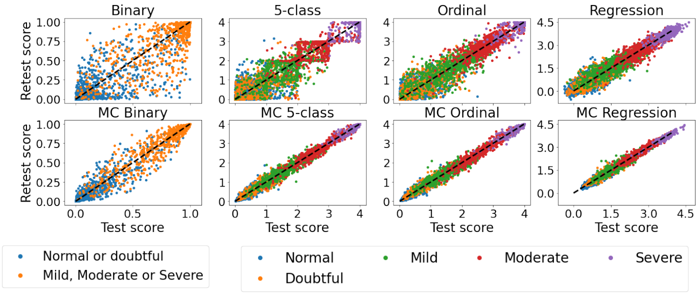

(a) Knee osteoarthritis classification

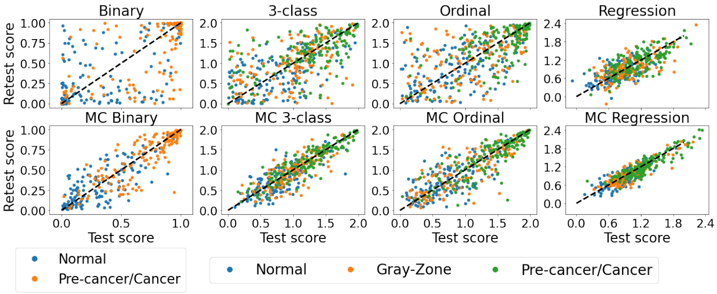

(b) Cervical classification

Supplementary Figure 1: **Comparison of severity scores obtained on different images, test and retest, from the same patient taken during the same visit.** Each data point is a pair a severity score from test retest images. When more than two images were available, the pair with the largest difference was retained. The dash line represents the identity line where the image pair are expected to be since the severity score from both images should have the same value.
